# Relationship between parenting stress and informant discrepancies on symptoms of ADHD/ODD and internalizing behaviors in preschool children

**DOI:** 10.1371/journal.pone.0183467

**Published:** 2017-10-10

**Authors:** Yu-Chi Chen, Shoou-Lian Hwang-Gu, Hsing-Chang Ni, Sophie Hsin-Yi Liang, Hsiang-Yuan Lin, Chiao-Fan Lin, Yu-Han Tseng, Susan Shur-Fen Gau

**Affiliations:** 1 Division of Clinical Psychology, Graduate Institute of Behavioral Sciences, College of Medicine, Chang Gung University, Tao-Yuan, Taiwan; 2 Department of Child Psychiatry, Chang Gung Memorial Hospital at Linkou, Tao-Yuan, Taiwan; 3 Department of Psychiatry, National Taiwan University Hospital and College of Medicine, Taipei, Taiwan; 4 Department of Psychology, Soochow University, Taipei, Taiwan; 5 Graduate Institute of Brain and Mind Sciences, College of Medicine, National Taiwan University, Taipei, Taiwan; 6 Graduate Institute of Clinical Medicine, College of Medicine, National Taiwan University, Taipei, Taiwan; University Children's Hospital Tuebingen, GERMANY

## Abstract

Parent and teacher ratings of child behaviors are often discrepant, and these discrepancies may be correlated with parenting stress. The present study explored whether various parenting stress factors are associated with discrepancies between parent and teacher ratings of attention-deficit/hyperactivity disorder and oppositional defiant disorder (ODD) as well as internalizing symptoms in preschool children. We recruited 299 Taiwanese preschool children (aged 4–6 years) from the community or via clinical referrals. A structural equation modeling was used to analyze the relationships among three factors derived from the Parenting Stress Index-Short Form and informant discrepancies on symptoms of inattention, hyperactivity/impulsivity, ODD, and internalizing behaviors. Scores reported by parents were higher for each of the symptoms examined than those reported by teachers, and the degree of agreement between informants ranged from low to moderate. The parental distress factor of parenting stress was associated only with parent ratings, whereas other factors of parenting stress—parent-child dysfunctional interaction and parents’ stress resulted from their child’s temperament—were correlated with both parent and teacher ratings. Only parental distress factor predicted informant discrepancies for all behavioral symptoms assessed. Our findings suggest that parental distress should be considered when parent rating scores show significant discrepancies from that of teacher rating scores.

## Introduction

Attention-deficit/hyperactivity disorder (ADHD) is a common neurodevelopmental disorder affecting about 5% of children [1] and 2.5% of adults [1] in most cultures. Around half of these children with ADHD also suffer from oppositional defiant disorder (ODD) [1]. Moreover, ADHD and ODD are the most common childhood mental disorders [2, 3]. According to the diagnostic criteria set out in the fifth edition of the Diagnostic and Statistical Manual of Mental Disorders [1], inattention or hyperactivity/impulsivity symptoms should be apparent in at least two settings. Parents and teachers are recognized as the key informants to report children’s ADHD symptoms and associated impairment in the home and school settings [4–6]. However, correlations between these two informants range from low to moderate for ADHD symptom ratings [6–8] as well as ODD symptoms [9, 10]. Furthermore, when using broadband psychopathological measurement scales such as the Child Behavior Checklist (CBCL), correlations between parent and teacher rating scores are also low to moderate for not only externalizing behaviors but also internalizing behaviors [11, 12]. Parent-teacher agreement between different informants is typically lower for internalizing behaviors than for externalizing behaviors [7, 12, 13] because the latters are easier to be observed [7, 14]. Moreover, scores rated by parents may be significantly higher than that rated by teachers [13, 15]. Many studies have demonstrated that ADHD symptoms are often present before children reach school age [16, 17]. Due to the limited development of cognition in preschool children, the assessment of ADHD symptoms mainly relies on parent or teacher reports [18]. A study conducted across 15 different regions also revealed discrepancies between parent and teacher rating scores among preschool children for most of the areas surveyed [15]. Thus, elucidating the reasons for informant discrepancies among preschool children across a wide range of symptoms may provide a great deal of valuable information for clinical practice.

Some have argued that discrepancies between parent and teacher symptom ratings can be attributed to setting differences (i.e., home versus school; [7, 9, 19, 20]). However, there may still be only a moderate correlation between different informant evaluations even when the informants see the index case in the same setting [21, 22]. Apart from the issue of different contexts, interpretations of behaviors may vary across informants, and informants might also have different thresholds at which they perceive or judge children’s behaviors as problematic [14]. Consequently, informants may provide different accounts even when the same behaviors are observed [23]. Although both parent and teacher ratings might be biased to a certain degree, several studies indicate that teacher ratings might be more accurate than parent ratings [9]. Because parents have fewer experiences and opportunities to compare their children’s behaviors with others’ [18], or they might expect that their children have well-behaviors [24], they might focus on their children’s minimal behavioral problems [18]. In contrast, teachers have a larger and more diverse samples for comparison [9]. Furthermore, parenting stress and parental depression may decrease the threshold at which parents determine that children’s behaviors warrant intervention, resulting in a wider gap between their rating scores and those of the child’s teachers [14]. In addition, some studies have shown that parenting stress has a greater impact on informant discrepancies in ratings of ADHD/ODD symptoms or broadband externalizing behaviors than does informant depressive symptoms [5, 6, 25].

Parenting stress refers to the stress perceived by parents in performing their role as parents and during parent-child interactions [4]. Parents of children with ADHD perceive significantly more stress than do parents of children without ADHD [21, 26]. The Parenting Stress Index-Short Form (PSI-SF), developed by Abidin [4], is one of the most widely used tools to measure parenting stress in parents of children with ADHD [27–29]. The PSI-SF is comprised of three factors [4]: parental distress, parent-child dysfunctional interaction, and difficult child. These parenting stress factors may be positively related to children’s behavioral symptoms. For example, DuPaul et al. [21] found that parents of preschool children with ADHD reported significantly higher levels of all three parenting stress factors compared with parents of preschool children without ADHD. These three parenting stress factors were positively associated with both externalizing and internalizing behaviors [30]. Rogers, Wiener, Marton, and Tannock [31] also found that parental distress was associated with both inattention and hyperactivity/impulsivity symptoms. Despite evidence for associations between parental stress and child emotional and behavioral problems, no study has further investigated the relationships between the individual parenting stress factors in PSI-SF and informant discrepancies in reporting child’s behavioral symptoms. However, identifying specific factors related to informant discrepancies is crucial because clinicians could refer to particular parenting stress domains rather than to the total parenting stress score when tailoring assessment information, or when selecting an intervention.

According to the definition of parenting stress [4], the parental distress factor might be predominately related to parents’ own cognitive and emotional reactions to parenting, whereas the parent-child dysfunctional interactions and the difficult child factors might involve those factors related to children’s behavioral problems. Therefore, it seems likely that different parenting stress factors might demonstrate differential relationships with informant discrepancies. Studies have shown that the effect of caretaker distress is significantly greater on the parent than teacher reports of children’s conduct and emotional problems [32]. Moreover, parental distress is positively correlated with parent ratings of ODD among preschoolers, with no such relationship for teacher reports [28]. In addition, parenting self-efficacy, which is negatively related to parental distress [33], shows a negative relationship with parent-teacher discrepancies on broadband externalizing behaviors, including ADHD/ODD [5]. Finally, many studies have suggested that parental depression, associated with parental distress, influences the parental evaluation of children’s emotional or behavioral problems, working to increase the discrepancies between parents and teachers when rating these behaviors [14, 34, 35]. Taken together, all these findings suggest that parental distress may distort parent ratings and is therefore closely related to informant discrepancies. Although the potential influence of the parent-child dysfunctional interaction or difficult child factor on informant discrepancies is still unclear, factors associated with the parent-child dysfunctional interaction, such as the relationships between parents and preschoolers, are not related to parent-teacher discrepancies for externalizing behaviors [5]. In addition, parent-child relationships do not predict father-mother discrepancies for either internalizing or externalizing behaviors [36]. Above studies may imply that the association between parent-child dysfunctional interaction and informant discrepancies is likely to be weak. Child’s temperament, a factor significantly related to the difficult child factor, is comparatively stable across contexts and development [37], and prior work suggests that child’s temperament factors do not predict teacher-parent discrepancies regarding ODD symptoms in preschool children [10]. Therefore, the relationship between difficult child and informant discrepancies may be relatively weak as well.

To examine whether different parenting stress factors may show different relationships with informant discrepancies on a broad range of emotional and behavioral problems, we employed the broadband CBCL to assess extensive psychopathology. However, because externalizing behaviors from the preschool version of the CBCL primarily measures attention problems and aggressive behaviors, its evaluation scope largely overlaps with ADHD/ODD. Therefore, the present study only included the internalizing behaviors from the CBCL, together with ADHD/ODD symptoms measured by the Swanson, Nolan, and Pelham Version IV Scale (SNAP-IV).

No studies have investigated the relationships between the distinct PSI-SF factors (parental distress, parent-child dysfunctional interaction, and difficult child) and informant discrepancies, particularly for preschoolers. Furthermore, parental distress-related factors, such as parental depression [14, 34, 35] and parenting self-efficacy [5], are closely related to informant discrepancies, while parent-child dysfunctional interaction-related factors, such as the child-parent relationship, may demonstrate a weak or no relationship with such discrepancies [5, 36]. Furthermore, because temperament is relatively stable across different settings, difficult child may also be a weak predictor of such discrepancies at best. We thus propose two hypotheses. First, we hypothesized that there would be discrepancies between parent and teacher ratings of inattention, hyperactivity/impulsivity, ODD, and internalizing behaviors among Taiwanese preschoolers. Second, we hypothesized that only parental distress would predict informant discrepancies on the aforementioned symptoms, with no such predictive effects from parent-child dysfunctional interaction or difficult child.

## Materials and methods

### Participants

Researchers distributed questionnaires to teachers and parents of 398 preschool children. Of these, 299 (75.1%) preschool children’s raters, both teachers and parents completed the whole questionnaires. The rest of the participants were excluded because of their teachers were unwilling to complete the entire questionnaires. However, an independent *t*-test on parent reports indicated that there were no significant differences between the two kinds of participants (all p-values > 0.05) for variables under the study. The study sample comprised 197 boys (59%) and 102 girls (41%) aged 4–6 years (mean age = 57.87 months, standard deviation = 7.6; see Table 1) recruited either from clinical referrals or from community kindergartens. Among them, 69 preschool children at risk for ADHD were referred by child psychiatrists from the National Taiwan University Hospital, Taipei, as well as the Chang Gung Memorial Hospital in Linkou; and 230 preschoolers recruited by convenience sampling from eight kindergartens located in New Taipei City and Kaohsiung City, Taiwan. Preschoolers who had schizophrenia, bipolar disorder, neurological diseases, and pervasive developmental disorders were excluded from the study. More than 71.2% of the parents had bachelor’s degrees or higher education. Moreover, similar to previous studies (e.g., [5]), the majority of questionnaires were completed by mothers (n = 253, 84.6%) because mothers usually are the main caregiver of preschool children [38], and are more involved in daily care of their children than fathers [31]. Since not all of the reports were completed by mothers, in this paper, we used the term “parents” rather than “mothers” as the informants. Furthermore, a dependent *t*-test (all *p values* > 0.05) revealed that mothers and fathers did not give significantly different ratings for any of the variables under the study.

**Table 1:** Sample characteristics.

	N = 299	
	Mean or Number	SD or %
Age (months)	57.87	7.60
Sex (male)	179	59.9%
Mother/Father		
Age	35.96/38.89	4.14/5.46
Education		
College and above	222/213	74.2%/71.2%
Senior high school	76/82	25.4%/27.4%
Occupation		
Professional	22/37	7.4%/12.7%
Skilled work	145/231	49.0%/79.4%
Other	128/23	43.2%/7.9%
Teacher		
Age	38.04	8.71
Teaching experiences (years)	13.75	8.45
Teaching the child (months)	8.66	7.45
Parenting Stress	72.78	19.16
Parental distress	27.60	7.99
Parent-child dysfunctional interaction	16.77	5.44
Difficult child	28.41	9.56

### Procedure

The present study was registered at ClinicalTrials.gov (NCT02433145). We began recruiting participants after receiving the approval from the research ethics committees (Research Ethics Committee of National Taiwan University: 201405086RINB; Research Ethics Committee of Chang Gung Memorial Hospital: 102-4775B). The purposes of the research and the research procedures were explained to the preschoolers’ parents and their teachers, and the informants were asked to sign an informed consent form. We also obtained the child assent from the preschool participants. After determining that each of the participants met the inclusion and exclusion criteria pertaining to age, medical conditions, and cognitive functioning, we asked the parents to complete the SNAP-IV, the CBCL, and the PSI-SF; and the teachers to complete the SNAP-IV and Caregiver-Teacher Report Form of the CBCL (C-TRF). After finishing the survey, each informant was given 100 New Taiwan dollars (approximately three US dollars) and a feedback letter that included the test results and relevant suggestions.

### Measures

#### The Chinese version of the SNAP-IV

The SNAP-IV scale contains 26 items that are each rated on a four-point Likert scale ranging from 0 (not at all) to 3 (very much). According to the diagnostic criteria set out in the DSM-IV-TR [39], this scale is divided into three subscales: inattention (9 items), hyperactivity/impulsivity (9 items) and ODD (8 items) symptoms [40]. The norms and psychometric properties for the Chinese version of the SNAP-IV have already been established for parents [40] and teachers [41] in Taiwan, and this scale has been widely adopted in child and adolescent research in Taiwan [42, 43]. Therefore, we utilized SNAP-IV to collect inattention, hyperactivity/impulsivity, and ODD symptom scores. In this study, the Cronbach’s alphas for each of the three subscales, ranging from 0.91–0.92 and 0.94–0.95, for parents and teachers respectively. These values demonstrate good internal consistency for both parents and teachers.

#### The Chinese version of the CBCL/1.5–5 and C-TRF

The Chinese version of the CBCL/1.5–5 and C-TRF scales have been widely used for assessing a range of emotional and behavioral problems in children aged 1.5–5 years based on parent and teacher reports, respectively [44, 45]. Each item of the two 99-item report form was rated on a three-point Likert scale ranging from 0 (not true) to 2 (very true or often true). Two categories are included: externalizing behaviors and internalizing behaviors, and we only employed the latter in the present study.

Several items differ across the CBCL/1.5–5 and C-TRF. Therefore, we included only those items that are identical in both versions (30 items assessing internalizing behaviors) in our analysis, to avoid the possibility that different items across versions could influence informant discrepancies [22, 46]. The Cronbach’s alpha for the shared internalizing behavior items was 0.91 for both the CBCL/1.5–5 and C-TRF, indicating that the selected items demonstrated good internal consistency for both parents and teachers.

#### The Chinese version of the PSI-SF

The present study used the Chinese version of the PSI-SF by Jen [47], which is derived from Abidin’s version [4]. The original Parenting Stress Index (PSI) comprises two main parts: The parent and child domains [48]. However, Solis and Abidin [49] have argued that merely considering the individual characteristics of parents and children is insufficient to explain parenting stress. With an emphasis on taking parent-child interactions into consideration, the PSI-SF comprises three factors: (1) Parental distress, which refers to the distress experienced by parents when fulfilling their parenting roles, and entails the following factors: perception of incompetence in parenting, feeling restricted in the parenting role, lack of social support, and the presence of depression [4, 50]; (2) Parent-child dysfunctional interaction, which refers to stress resulted from the disappointment that parents experience during their interactions with their children [4]; and (3) Difficult child, which refers to the stress which parents perceived from the temperament or behavioral characteristics of their children which are difficult to manage or care for [4]. This version has demonstrated excellent reliability and validity [44, 47]. The 34 items are rated on a five-point Likert scale ranging from 1 (strongly agree) to 5 (strongly disagree) and include 11 parental distress items, ten parent-child dysfunctional interaction items, and 13 difficult child items. The PSI-SF addresses the issue of the amount of time required to complete the 101-item long-form PSI and is thus more suitable for screening purposes [4]. In this study, the Cronbach’s alpha value of the total scores is 0.93, and those for each of the three subscales ranged from 0.87–0.91, indicating the good internal consistency of this scale. We carried out confirmatory factor analysis (CFA) to verify a three-factor structure. The following results were obtained: Normed chi-square (NC2) value = 1.33 (< 3; [51]), root mean square error of approximation (RMSEA) = 0.033 (< 0.05, [52]), standardized root mean square residual (SRMR) = 0.020 (< 0.05; [53]), comparative fit index (CFI) = 1.00 (> 0.95; [54]), goodness-of-fit index (GFI) = 0.98 (> 0.90; [55]). These values indicate that the three-factor model of the PSI-SF is supported.

### Data analysis

Many studies suggest that ADHD can be treated as a continuous behavioral trait [19, 56]. In this study, we followed the methods of Theule et al. [28] and Rogers et al. [31] to analyze the entire pool (community and clinical referral) of preschool children’s behavioral observations dimensionally, to maximize statistical power. There was only one missing data in the study sample, and this missing value was replaced by the item mean. Dependent *t*-tests were performed to assess whether parent and teacher total raw scores on the inattention, hyperactivity/impulsivity, and ODD subscales of the SNAP-IV as well as total raw scores for the internalizing behavior items on the CBCL and the C-TRF, differed significantly. The Pearson correlation was used to evaluate agreement between parents and teachers. The current study utilized Holm [57]'s step-down procedure to adjust *p* values of the dependent *t-*test and Pearson correlation test.

The present study used standardized difference scores (SDS) for subsequent measurements of informant discrepancies, calculated by converting each informant raw scores to *z*-scores and then deducting the teachers’ *z*-scores from the parents’ *z*-scores [6, 57]. SDS are appropriate when conducting research on the relationship between informant discrepancies and informant characteristics [58]. We used the total, inattention, hyperactivity/impulsivity, and ODD of the SNAP-IV, total CBCL/1.5–5 and total C-TRF scores on shared internalizing behavioral items to calculate SDS.

The Pearson correlations were subsequently used to analyze the relationships between parenting stress and its factors (parental distress, parent-child dysfunctional interaction, and difficult child) with (1) parent and teacher rating scores as well as (2) SDS for inattention, hyperactivity/impulsivity, ODD, and internalizing behaviors. Furthermore, we used LISREL 8.7 [59] to carry out structural equation modeling (SEM), simultaneously analyzing the relationships between parenting stress factors and symptom rating discrepancies. Most previous studies of the relationship between informant characteristics and informant discrepancies have applied regression analysis [6, 10, 22]. However, Lavigne et al. [8] argued that SEM allowed for a way to determine the relative difference in the magnitude of the relationship between a variable and parent and teacher reports of ADHD. Moreover, SEM can be used to simultaneously compare each of the possible predictors [60], as well as to evaluate whether biases are due to random error or whether they are indeed caused by construct-related variations [61]. The present study used SEM to assess whether the aforementioned three parenting stress factors predict informant discrepancies on reports of ADHD/ODD and internalizing behaviors. Moreover, many behaviors in ADHD are similar to those seen in depression and anxiety (with belong to the internalizing behavior dimension; [1]), so we examined inattention, hyperactivity/impulsivity, ODD and internalizing behavior all within a single model for analysis. We used the item parcel method to parcel items of each subscale into three indicators. This approach has been widely used [62–64], as it has high reliability and yields more stable parameter estimation results [65], in addition to generating a better model goodness-of-fit [66]. We followed the recommendations of Little et al. [58], randomly collecting the items for each subscale into three parcels to serve as indicators. This item parcel approach included the PSI-SF items as well as the aforementioned SDS scores for the SNAP-IV, CBCL, and C-TRF. We performed CFA on the PSI-SF, following the item parcels [64, 67]. According to Joormann and Stöber [67], carrying out CFA after item parceling produces more precise estimation results compared to the use of individual items, because parcels demonstrate higher reliability and fewer parameters than do individual items. Finally, model goodness-of-fit was assessed using several indices. Model fit is deemed to be excellent with an NC2 value of less than 3 [51], an RMSEA value of less than 0.05 [52], an SRMR of less than 0.08 [53], a CFI greater than 0.95 [54], and a GFI of greater than 0.90 [55].

## Results

Parent total rating scores for IA, HI, ODD, and internalizing behaviors were all significantly higher than corresponding teacher scores (*t*s(298) = 2.38–10.53, *p*s < 0.001, despite the small (Cohen’s *d* = 0.13, inattention) to moderate (Cohen’s *d* = 0.62, ODD) effect sizes the correlations between informants ranged from low to moderate (*r*s = 0.22–0.57, *p*s < 0.001; Table 2).

**Table 2:** Parent and teacher rating scores.

	Parents	Teachers	Dependent *t*-test	Cohen’s *d*	Parents-Teachers Correlation
	Mean (SD)	Mean (SD)			
Inattention	7.99(5.66)	7.16 (6.83)	2.38*	0.13	0.56**
Hyperactivity/impulsivity	8.66 (6.54)	6.11 (7.24)	6.88**	0.37	0.57**
Oppositional defiant	7.60 (5.18)	3.93 (5.58)	10.53**	0.66	0.37**
Internalizing	9.61 (8.06)	5.45 (6.86)	7.69**	0.56	0.22**

Note

*p< 0.05

**p < 0.001, all *p* values are adopted by Holm's step-down procedure

Parenting stress total scores were significantly related to both parent and teacher ratings of inattention, hyperactivity/impulsivity, ODD, and internalizing behaviors (*r*s = 0.25–0.53, *p*s < 0.001; Table 3). Furthermore, correlations between parenting stress factors (parental distress, parent-child dysfunctional interaction, and difficult child) and parent/teacher ratings revealed that both parent-child dysfunctional interaction and difficult child were significantly related to both informant ratings (*rs* = 0.15–0.56, *ps* = 0.040–< 0.001). On the other hand, although parental distress was significantly associated with parent ratings *(rs* = 0.29–0.33, *ps* < 0.001), parental distress was unrelated to teacher ratings (*rs* = 0.06–0.09, *ps* = 0.54).

**Table 3:** Correlation matrix for parenting stress and symptom ratings.

Parenting stress	Inattention	Hyperactivity/ impulsivity	Oppositional defiant	Internalizing
Total score	0.51**/0.28**	0.49**/0.25**	0.46**/0.25**	0.53**/0.27**
Parental distress	0.29**/0.06	0.33**/0.09	0.30** /0.08	0.33**/0.08
Parent-child dysfunctional interaction	0.40**/0.26**	0.28**/0.15*	0.23**/0.17*	0.41**/0.23**
Difficult child	0.55**/0.36**	0.55**/0.35**	0.55**/0.35**	0.56**/0.33**

Note

*p < 0.05

**p < 0.001, all *p* values are adopted by Holm's step-down procedure

*Note*: Value before the forward slash is the correlation coefficient for parental stress and parents’ ranting scores; the value after this is the correlation coefficient for parental stress and teacher rating scores.

The correlation matrix for parenting stress and informant discrepancies showed that parenting stress total scores were significantly associated with inattention, hyperactivity/impulsivity, ODD, and internalizing behaviors SDS (*rs* = 0.19–0.26, *ps* = 0.008–< 0.001; Table 4). Parental distress and difficult child factors were significantly associated with the SDS of the four subscales (*r*s = 0.18–0.26, *ps* = 0.006– < 0.001), while parent-child dysfunctional interactions were not significantly related to the SDS of the four subscales (*r*s = 0.06–0.14, *ps* = 0.0512).

**Table 4:** Correlation matrix for informant discrepancies and parenting stress.

	1	2	3	4	5	6	7	8
1. Inattention	1.00							
2. Hyperactivity/impulsivity	0.62***	1.00						
3. Oppositional defiant	0.43***	0.66***	1.00					
4. Internalizing behaviors	0.43***	0.45***	0.56***	1.00				
5. Parenting stress total score	0.25***	0.26***	0.19**	0.21**	1.00			
6. Parental distress	0.25***	0.26***	0.20**	0.20**	0.79***	1.00		
7. Parent-child dysfunctional interaction	0.14	0.13	0.06	0.14	0.80***	0.46***	1.00	
8. Difficult child	0.21**	0.22**	0.18*	0.18*	0.89***	0.49***	0.65***	1.00

Note

*p < 0.05

**p < 0.01

***p < 0.001, all *p* values are adopted by Holm's step-down procedure

*Note*: Inattention, hyperactivity/impulsivity, Oppositional defiant, and internalizing behaviors are all standardized difference scores (SDS)

**Fig 1:**
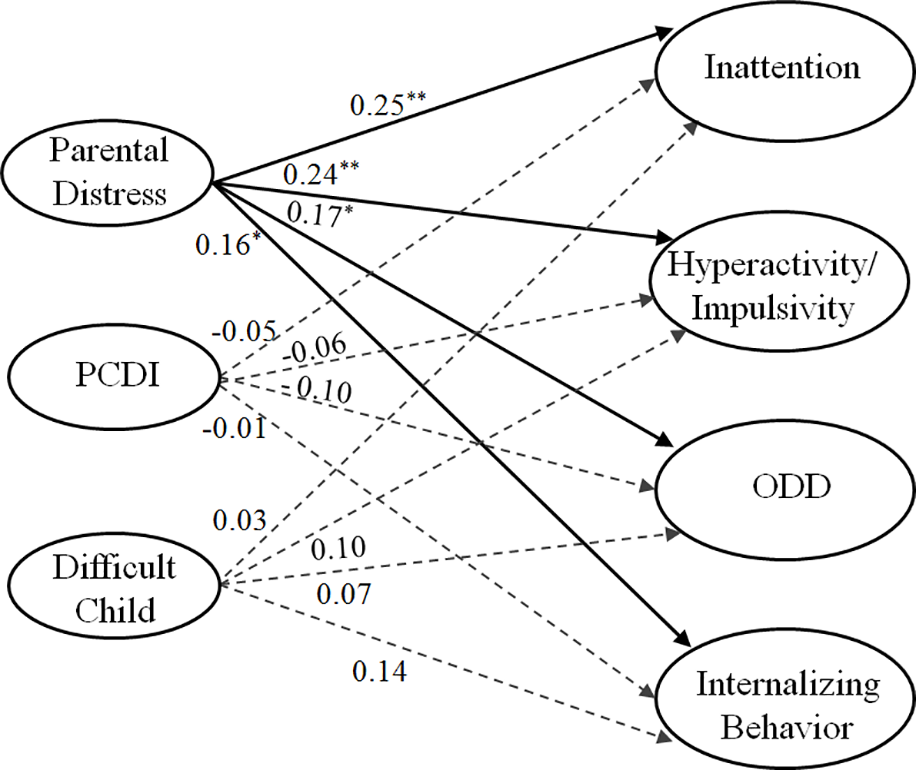
Model of effects of parenting stress factors and informant discrepancies on symptoms of ADHD/ODD and internalizing behaviors. PCDI denotes parent-child dysfunctional interaction; ODD denotes oppositional defiant disorder. ^*a*^*Note*: Parental Distress, parent-child dysfunctional interaction, and Difficult Child are associated with one another; Inattention, Hyperactivity/Impulsivity, ODD, and internalizing behaviors are associated with one another. ^*b*^*Note*: Inattention, hyperactivity/impulsivity, Oppositional defiant, and internalizing behaviors are all standardized difference scores (SDS).

Finally, we used SEM in order to confirm the relationships between parenting stress factors and informant discrepancies on behavioral symptoms, as well as to verify the results of the correlation analysis. A model in which parenting stress predicts informant discrepancies provided a good fit for the data, with NC2 = 1.33 (< 2), RMSEA = 0.033 (< 0.05), SRMR = 0.033 (< 0.08), CFI = 0.99 (> 0.95), and GFI = 0.93 (*p* > 0.90). The model results (see Fig 1) indicate that the parental distress factor significantly predicts informant discrepancies for inattention (*γ* = 0.25, *p* = 0.002), hyperactivity/impulsivity (*γ* = 0.24, *p* = 0.003), ODD (*γ* = 0.17, *p* = 0.033), and internalizing behavior (*γ* = 0.16, *p* = 0.037). However, as hypothesized, parent-child dysfunctional interaction and difficult child did not show significant effects on informant discrepancies for any of the symptom ratings (*p*s = 0.175–0.936).

## Discussion

The present study is the first to investigate the specific relationships between the different parenting stress factors that comprise the PSI/SF and parent-teacher discrepancies in reporting behavioral symptoms in preschoolers. We found that the average scores reported by parents for inattention, hyperactivity/impulsivity, ODD, and internalizing behaviors were significantly higher than those reported by teachers, with only low to moderate correlations between informants. Moreover, while parental distress was positively associated with parent rating scores for each behavioral symptom domain, this factor was unrelated to teacher rating scores for any symptom domain. However, parent-child dysfunctional interaction and difficult child were significantly associated with the rating scores of both parents and teachers for all symptoms assessed. Finally, SEM analysis revealed that the parental distress factor predicts informant discrepancies for all symptoms, whereas the parent-child dysfunctional interaction and difficult child factors have no such predictive power.

### Parent and teacher rating scores are discrepant

The present study demonstrated that parents and teachers often provide discrepant ratings of child behaviors. Similar to previous studies, parents provided higher symptom ratings than teachers for inattention, hyperactivity/impulsivity [18, 24], and externalizing as well as internalizing behaviors [25] among preschoolers. Despite the very small effect size, the differences between parent and teacher ratings for inattention were significant. Re and Cornoldi [18] also reported that the parent-teacher discrepancy in inattention is smaller than that in hyperactivity/impulsivity. The reason for this discrepancy could be that inattention is comparatively less common in both parents’ and teachers’ ratings at this age [68] because there are fewer attention-demanding assignments for preschoolers. Moreover, the correlations between parent and teacher ratings ranged from low to moderate, which is also in line with previous studies [7–10, 13, 15]. One study focused on school-aged children among different countries across Western and Eastern regions showed that low correlations for both externalizing and internalizing behaviors between parent and teacher ratings, with generally higher scores based on parent reports [13], and the correlation between scores rated by parents and teachers are also low for preschool children across a variety of countries [15]. This phenomenon seems to emerge across different cultural groups and both preschool and school-aged children, for the reason that behavioral symptoms are variable across different settings.

### Parental distress as the only PSI/SF factor to predict informant discrepancies

Our correlation results showed that parenting stress total scores were significantly related to informant discrepancies on inattention, hyperactivity/impulsivity, ODD, and internalizing behaviors. Our results support the finding of van der Oord et al. [6] that parenting stress is significantly associated with parent-teacher discrepancies on inattention, hyperactivity/impulsivity, and ODD. It is also consistent with the findings of Gross et al. [5] and Langberg et al. [22], who have reported that parenting stress may be associated with informant discrepancies. It is possible that parenting stress may increase parent-teacher discrepancies by decreasing a parent’s judgment threshold at which a child’s behavior is considered problematic [14]. However, these prior studies did not examine the predictive powers of the separate parenting stress factors. The present study is the first to identify the specificity of the relationships between the different PSI/SF factors and informant discrepancies on ADHD/ODD symptoms and internalizing behavior in preschool children.

Although parent-child dysfunctional interaction and difficult child were significantly associated with both parent and teacher ratings, parental distress was related only to parent ratings. Given that the correlations between total parenting stress scores and discrepancies serve to obscure such relationship in a more specific pattern, our findings of only parental distress specifically driving the relationship between parenting stress and informant discrepancies based on SEM analyses. These findings indicate that parenting-related distress plays the key role in such discrepancies, with specific parent-child interactions or child temperament relegated to a peripheral role.

Although few studies have investigated the effect of parental distress on informant discrepancies between teachers and parents regarding preschool children, one study of children aged 2–4 years demonstrated that parenting self-efficacy, which is negatively related to parental distress [33], was negatively associated with parent-teacher discrepancies on externalizing behaviors [5]. In addition, studies of school-aged children have also shown that parental distress is significantly associated with parent but not teacher ratings on ODD [28]. Similarly, caregiver distress has a greater effect on parent reports than teacher reports of the emotional problems on these children [32]. Furthermore, researchers have mentioned the possibility of bidirectional relationships between parenting stress and the severity of children’s symptoms [69, 70]. However, our results demonstrate that parental distress may have such a bidirectional relationship only with the severity as perceived by parents, which indicates that parental distress may bias only parent evaluations, thereby leading to informant discrepancies.

As expected, we found that neither parent-child dysfunctional interaction nor difficult child significantly predicted informant discrepancies, which may be the possible reason that both parent-child dysfunctional interaction and difficult child are related to child behaviors, as compared to parental distress which is more related to parents’ own emotional issues than child factors. Our findings here are in line with a previous finding that parent-child relationships, related to parent-child dysfunctional interaction, were not associated with parent-teacher discrepancies on externalizing behaviors in preschoolers [5]. Treutler and Epkins [36] found that parent-child relationship among school-aged children failed to predict father-mother discrepancies on the ratings of externalizing as well as internalizing behaviors. Furthermore, our finding of moderate correlations between difficult child with parent and teacher rating scores of behavioral symptoms lend evidence to support our hypothesis that parents and teachers have similar perceptions of a given child’s temperament (e.g., as “difficult to handle”). Such similar perceptions should not be associated with appreciable discrepancies in ratings. Finally, our results are also consistent with the findings of Lavigne et al. [10], who concluded that temperament factors do not predict teacher-parent discrepancies regarding ODD symptoms in preschool children.

### Implications for practice and research

First, because there is currently no “gold standard” regarding who can provide absolutely accurate information about child behaviors [14, 71], clinicians and researchers face with having to combine reports from different sources. However, informant discrepancies increase the difficulty of defining the presence of emotional/behavioral problems or making a diagnosis. Despite this, informant discrepancies should be viewed as an opportunity to gather data in clinical settings [14]. Clinicians and researchers can gather more information to evaluate the credibility of different informant reports and then integrate this information [72]. For example, the availability and utilization of information on parenting stress, particularly parental distress, would benefit diagnosis and intervention. That is, if large discrepancies exist between the scores reported by parents and teachers, and the parents also report obviously higher parental distress scores, it should be considered whether parent reports are unduly influenced by parental distress. Moreover, parental distress sub-questionnaire could be viewed as a quick survey of the reasons for the discrepancies. Clinicians could further investigate the possible factors, such as parental depression, on the basis of parent answers to the parenting distress sub-questionnaire, or consider referring the parents to appropriate professional assistances. Correspondently, each parenting stress factor could be read as valuable additional information for developing the specific intervention. Clinical intervention approaches may differ according to different kinds and severity levels of parenting stress factors. For example, with increased severity of parental distress, individual treatment may be required for parents; with the presence of a difficult child, training in child behavior management may be required [73]. Previous interventions with parents have emphasized training in parenting skills and parent-child interactions (e.g., behavioral parent training; [74]). Based on our results, we further recommend that parental distress-related factors be included in treatment programs, such as strengthening parenting self-efficacy, instructing parents in realistic expectations as well as positive thinking, and providing timely referrals for parents undergoing emotional reactions such as depression. In addition, support or self-help groups for parents may be organized to increase parent social supports.

### Methodological limitations

This study has the following limitations. First, a single data collection time point limits any causal inference about the effect of parenting stress on informant discrepancies. Second, teacher ratings are generally considered to be more accurate than that of parent ratings because of their experience in observing groups of preschoolers [9, 18]. However, factors that might affect teachers’ ratings, such as teachers’ attitudes, stress, psychopathology, and whether the classes were structured [68] were not assessed in this study. It is suggested that these variables be included in future studies. Third, we did not formally assess major psychopathology such as depression, anxiety, and ADHD in the parents and teachers as well. These data warrant collection in future studies. Fourth, more data from fathers should be collected in future investigations, given increasing recognition of the paternal parenting role and an increased understanding of gender effects on parenting over the past decade.

## Conclusion

The present study provides evidence to support the view that parents tend to report more severe behavioral symptoms than do teachers in preschoolers. Moreover, parental distress, but not parent-child dysfunctional interaction or difficult child, predicts parent-teacher discrepancies in reports of behavioral symptoms in preschoolers. This new finding can be explained by the correlation between parental distress and the children’s symptom severity as perceived by parents, rather than those problems observed by teachers.

## Supporting information

S1 File PONE-D-17-00374-General-Data. (XLSX)
